# Development of a multi-use decision support system for scientific management and breeding of sheep

**DOI:** 10.1038/s41598-022-24091-y

**Published:** 2022-11-12

**Authors:** Ambreen Hamadani, Nazir A. Ganai

**Affiliations:** Sher-e-Kashmir University of Agricultural Sciences and Technology of Kashmir, Srinagar, India

**Keywords:** Genetics, Animal breeding, Immunogenetics, Inbreeding

## Abstract

As the challenges of food insecurity and population explosion become more pressing, there is a dire need to revamp the existing breeding and animal management systems. This can be achieved by the introduction of technology for efficiency and the improvement of the genetic merit of animals. A fundamental requirement for animal breeding is the availability of accurate and reliable pedigreed data and tools facilitating sophisticated computations. Keeping this in view, Smart Sheep Breeder (SSB) was developed using the waterfall methodology and multiple programming languages. It is a multi-use online artificial intelligence (AI) based and internet of things (IoT) compatible decision support system (DSS). It is capable of automatic performance recording, farm data management, data mining, biometrical analysis, e-governance, and decision-making in sheep farms. A centralized database was also developed capable of ranking sheep across multiple farms based on genetic merit and effective dissemination of germplasm. The system in India is available as a web-based tool and android application which facilitates performance recording and generates customized reports on various aspects of sheep production. SSB uses artificial intelligence and biometrical genetic algorithms to calculate breeding values, and inbreeding coefficients, construct selection indices and generate pedigree, and history sheets as well as more than 40 types of custom-tailored animal and farm reports and graphs. The algorithms used were validated using on farms using farm data and also by comparison with established methods and software. Smart Sheep Breeder could thus prove to be indispensable for the present farming systems which could be used by sheep farm managers and breeders across India.

## Introduction

The sheep husbandry sector is an important livelihood source for a significant percentage of people in India especially landless and marginalized farmers. There is an incessant demand for sheep and sheep products, be it in terms of mutton or wool. Despite this, the sector has been unable to reach its true potential and most animals are of poor genetic merit therefore the overall productivity of each farm is low. This is true even in parts of the country where there is huge scope for improving sheep husbandry. For example, Jammu, and Kashmir, a Union Territory in India has the 6th highest population of sheep and yet the genetic merit of the animals is reported to be declining over the years^1–5^.

The sluggish growth and development may be attributed to the lack of proper data recording, both at the farm and field level, and the low dissemination of modern technologies for storage, management, and analysis of farm data. This would otherwise have enabled farm managers to quickly comprehend the status of the farm and discover lacunas in managemental practices. Also, due to the lack of proper breeding tools and a failure to connect with sheep breeders for performance evaluation of their animals, sheep farm managers still rely more on their perception than on scientific methods of selection and breeding. This limits the scope for open nucleus breeding schemes (ONBS) for continuous genetic upliftment of the farms and farm flocks. As a result, the rate of genetic growth is slow and elite animals often end up in slaughterhouses and their genetic potential remains unutilized.

The adoption of digital technologies could turn the scenario around. The fourth industrial revolution has transformed the animal husbandry sector of developed countries and taking leaves out of the success stories like Sheep Genetics Australia^6^, could uplift the animal husbandry sector in India as well. There is a dire need for the development of decision support and data recording systems custom tailored for the specific needs of Indian farms which could potentially help in eliminating uncertainties, increasing profits, and improving the genetic merit as well as the welfare status of farm animals. The Information Network for Animal Productivity and Health (INAPH) for cattle in India is one such endeavor^7^.

However, no such program is currently in place for the complete management of sheep throughout the country. This is especially true for breeding tools. Scientific selection of animals is very cumbersome using conventional methodologies and because of the complexity involved, many farm managers either do not know how to compute it or do not possess enough computational power in their systems to do so. Due to this, the genetic potential of animals is largely unexplored, farm management is cumbersome and labor-intensive. All this results in fewer returns to the farmers. Isolated efforts for bringing digital technology into this sector could be traced in literature. A farm management information system for goats^8^ and other toolkits^9^ have been developed in India, but none provide comprehensive decision support. Such tools also do not assist in the scientific selection of animals on pure genetic merit.

Therefore, the present research was undertaken for the development of a multi-use online decision support system (DSS). This would (1) enable performance recording in sheep, (2) automate statistical estimation, (3) generate customized reports on various aspects of sheep production, (4) support efficient e-governance, and (5) help in the selection of superior animals. (5) Ranking animals on genetic merit through estimated breeding values for the effective selection of animals is yet another critical aspect of this tool. All this is critical for efficient farm management and sheep breeding.

## Materials and methods

### Design methodology

#### Hypothesis

It was hypothesized that farm data collection, analysis, and interpretation could be automated for the overall benefit and welfare of the animals, farms, and farm managers. The hypothesis that the entire process of breeding value estimation and ranking of animals based on genetic merit could be automated for identifying superior animals on the farms was also tested. The other questions that were identified were whether such e-services would be useful to the stakeholders.

#### Development methodology

**Figure 1:**
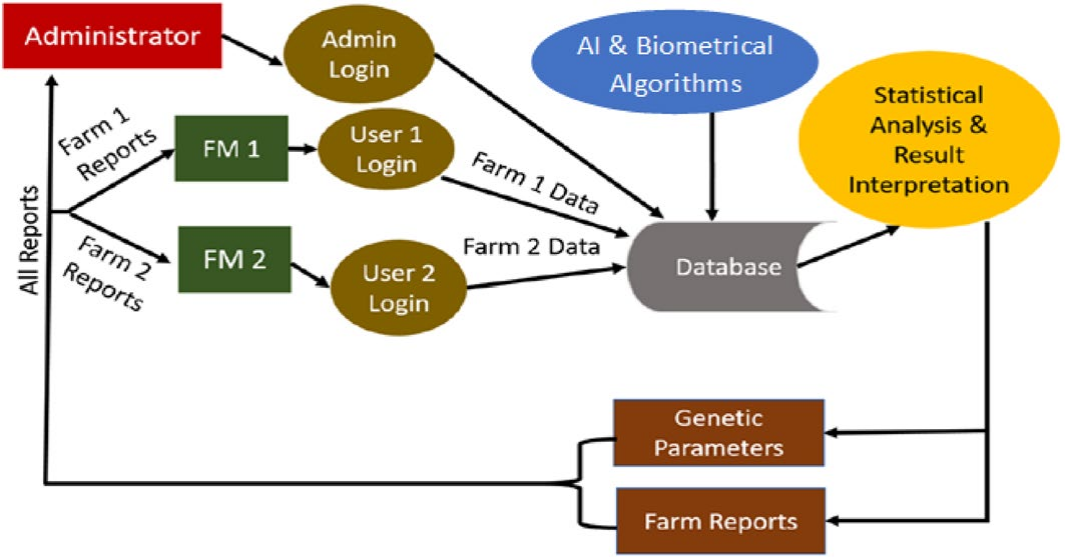
Data flow diagram level.

The software development methodology used for the design and development of the present DSS was the waterfall model. The waterfall model is a linear and sequential approach, and the following stages of development marked the development of this tool:

1. **Planning and requirement analysis** Identification of the requirements in animal farms was done by surveying at Mountain Research Station for Sheep and Goat (MRCSG), SKUAST-K, and other organized government sheep farms of J&K, India.
2. **Design** The design of the tool was created based on the requirements of the farms and entity relationship models (ER diagrams) and data flow diagrams (DFD) were constructed and reviewed based on various parameters and the best DFD (Fig. 1) was selected for the development of the tool.
3. **Development and deployment** The development, testing, and integration of software was done primarily on a Windows operating system and tested on Ubuntu operating system using the following programming languages:
  - HTML and CSS, JavaScript, and jQuery were used for frontend development and for creating e-forms which are discussed in the next section.
  - JAVA was used for developing the mobile application.
  - PHP, R, Python, MySQL, phpMyAdmin, and Django were used for developing more complex scripts involving biometrical algorithms discussed in detail in the later sections. Many libraries were utilized for building the tools: These include MCMCglmm, Pedigree, MasterBayes, MASS and GeneticsPed, DBI and RMySQL, proto, gsubfn, nlm, cronR were used for building the DSS^10–19^.
  - The system was hosted on a VPS or Virtual Private Server on Ubuntu 17.10 x64 operating system, and the android application was hosted on Google Play Store.
  - IoT Integration: The application programming interface (APIs) for the DSS was developed so that it could be integrated with IoT-enabled devices and tools and with android applications. Raspberry Pi microcontroller was used along with RFID tags, readers, and antennae for building the automatic weight recording system. The customization of an automated system for automatic animal identification, counting of animals, and automatic data capture of weight records was outsourced.
4. **Testing and validation** Both alpha and beta tests on the web-based tool were performed. System tests helped to validate the fully developed system to ensure it meets all requirements and functions accurately^20^. Alpha testing was performed by the development team to validate the tool by comparison with standard methods^21,22^. Testing was also done using data from 3 sheep breeds from four farms using real-time data as well as retrospective data. The retrospective data from 1969 to 2016 was collected from MRCSG, Shuhama for the Corriedale breed was collected. Data for Rambouillet was collected from Government Sheep Breeding Farm (GSBF), Reasi from 1998 to 2007. For Kashmir Merino, it was collected from GSBF, Kralpathri from 1997 to 2016, and from GSBF, Goabal from 2013 to 2016. The data were also used for the development and validation of the deep learning model.

Beta testing was also done by the officers/farm managers of the Sheep Husbandry Departments of Jammu and Kashmir who were given training about this tool. Their feedback was used for bug fixes, DSS evaluation as well as for evaluating the usefulness of the tool. Results were also compared with known software like Wombat^23^.

### E-forms and farm reports

To allow the end-user to input farm data into the DSS, e-forms were created both in the web-based as well as an android application to allow the end-user to add or edit all details about new animal records, body weights, body measurements, wool (yield and quality), treatment, mating/breeding, vaccination/dosing, castration, and disposal for their subsequent analysis and mining were written using PHP, R, Python, and Java. Data retrieval from the MySQL database was done using MySQL queries which were used for the creation of scripts for several types of comprehensive farm reports on all aspects of farm management.

### Biometrical and artificial intelligence methods

The decision support system was created in such a way that the breeder could be provided with tools to scientifically select sheep, requiring no prior knowledge of animal breeding. Scripts were, therefore, created for real-time data analysis and estimate generation. These mainly included the following.

#### Sib analysis for heritability estimation^24^

For the estimation of heritability (Eq. (1)), the Nested ANOVA methodology was used where full sibs are nested within half-sibs and

1 $$h^{2} = \frac{{\sigma_{A}^{2} }}{{\sigma_{p}^{2} }}$$

where, *σ*^2^*A* = additive genetic variance and *σ*^2^*p* = phenotypic variance. The sire component of variance equals 4*σ*^2^*s/σ*^2^*p,* dam component equals 4*σ*^2^*d/σ*^2^*p* and sire + dam components equals 2(*σ*^2^_*s*_ + *σ*^2^_*d*_)/*σ*^2^_*p*_*.*

#### Animal model for heritability estimation^25^

Automated scripts for heritability were also created using generalized linear mixed models. For the traits considered under the model, sex and year were taken as fixed effects. The animal effect, sire effect, and dam effects were taken as random to account for the possible bias caused by these effects. The effect of the farm was included under the condition of the availability of data from more than one farm. The code was kept flexible enough to include analysis breed-wise as well. This methodology was incorporated into the DSS, and scripts were created in such a way that the heritability estimated using this methodology could be used as a variable for the estimation of breeding values of all live animals on the farm in real-time.

#### Breeding values using BLUP

Script for the automatic estimation of breeding values was written based on individual selection^26^. The breeding values were estimated using Eq. (2).

2 $$Breeding\;Value = P_{m} + \, h^{2} \left( {P_{i} - \, P_{m} } \right)$$

where *P*_*m*_ = population means, *P*_*i*_ = phenotypic value of the individual, *h*^2^ **=** heritability. The model in the matrix and mixed model solutions for BLUP are given in Eq. (3)

3 $$Y = Xb \, + \, Zu \, + \, e$$

$$[X^{\prime}X\;X^{\prime}Z\;Z^{\prime}X\;Z^{\prime}Z + \lambda A^{ - 1} ] \quad [b\;u] = [X^{\prime}y\;Z^{\prime}y]$$

where, *Y* = selected trait, *b* = fixed vector for different non-genetic factors assumed to influence the traits, *u* = random vector for breeding values of sires to be predicted, *e* = random error, *X, Z* = incidence matrices, *λ* = (4*-h*^2^)*/h*^2^*, h*^2^ = heritability, A = numerator relationship matrix.

#### Selection indices

The DSS was developed to include selection indices for multiple user-defined traits. The selection index was constructed as a weighted sum of BLUP-EBV of selection^27^. The selection criterion, is, therefore, an index of known phenotypes or EBV, constructed so that the expected value of the index is equal to H. Selection index scripts were created using the formula given in Eq. (4).

4 $$I = v_{1} EBV_{1 } + v_{2} EBV_{2} + \cdots + v_{n} EBV_{n}$$

where *I* = index value, *EBV* = estimated breeding values for the breeding goal traits, *n* = number of traits, and *v*_*i*_ = (economic) weighting factors in the breeding goal. The code was written in such a way that the EBVs could be obtained from the estimation done using BLUP in the form of variables and was not kept as user input.

#### Relative economic values

Scripts for relative economic values of different traits were written based on prices of different commodities (meat and wool) prevailing in the market and were estimated as per Ganai^28^. Economic values of body weight at different ages were calculated as given in Eq. (5).

5 $$\frac{{\left( {\frac{Book\;Value\;of\;the\;Trait}{{Mean\;of\;the\;Trait}}} \right)}}{Days\;to\;Attain\;the\;Value} \times Survivability$$

The economic value for fleece weight was calculated as given in Eq. (6):

6 $$\frac{Book\;Value}{{Days\;to\;attain\;value}} \times Mean\;Fleece\;Weight$$

Scripts for economic values of fiber diameter and staple length were written. This required the use of simple regression for both traits to calculate the regression of price per kg of wool per unit of fiber diameter and staple length. Since the price changes as per the market conditions, scripts were written such that the values as input by the user could be used to calculate the regression coefficients and thereafter the relative economic values for wool quality traits as given in Eq. (7)

7 $$y = ax + b$$

‘*a*’ was calculated using the formula given in Eq. (8):

8 $$y - \overline{y} = a\;(x - \overline{x})$$

where, *y* = dependent variable (price), *x* = independent variable (staple length and fibre diameter), *a* = regression coefficient, b = constant, $\overline{y} = {\text{mean}}\;{\text{of}}\;y,\;\overline{x} = {\text{mean}}\;{\text{of}}\;x$.

#### Inbreeding coefficients

Scripts were written in R for the automatic calculations of inbreeding coefficients for individuals based on the pedigree data retrieved from the database. The formula for the inbreeding coefficient is given in Eq. (9)

9 $$FX = \, \sum \, \left[ {({1}/{2}){\text{n}} + {1}({1} + {\text{FA }})} \right]$$

where *FX* equals the inbreeding coefficient of *X* individual, n is the number of segregations between sire and dam in a path emanating from a common ancestor, *FA* is the inbreeding coefficient of the common ancestor. Inbreeding was estimated using the pedigree package in R.

#### Pedigree and history sheets

The script for the display of pedigree trees is written in PHP. Scripts for the history sheets were written to generate comprehensive information about the production and reproduction of the animal as well as its progeny.

### Artificial intelligence (AI) algorithms

Deep artificial neural networks (ANN) were used for developing a model for the prediction of breeding values. Estimated breeding values were used as labels for training and evaluating the neural network. The Django framework was used for building the framework for the AI module, which was deployed using TensorFlow Serving^29,30^. A variable number of features were used for training the network to arrive at the optimal model. The number of neurons, hidden layers, optimum learning rate, activation function, optimizer settings, etc. were determined heuristically. The data were normalized and cleaned before training.

## Experimental findings

### Planning and requirement analysis

A farm survey of organized sheep farms indicated that most of the data recording on farms was still based on paper records which were not consolidated, and negligible digitization had taken place so far. Centralized databases for data management were not present which led to complete data loss on one farm during a fire one. Breeding decisions were mostly based on intuition rather than scientific procedures and inferences considering all data at the farm for any date were not drawn. A mechanism for connecting farms with scientists also didn’t exist. Complete handy records for individual animals were not maintained and decisions related to sale/transfer/disposal/breeding were reported to be often based only on the most recent records of the animal and pedigree was not taken into consideration.

### DSS development

**Figure 2:**
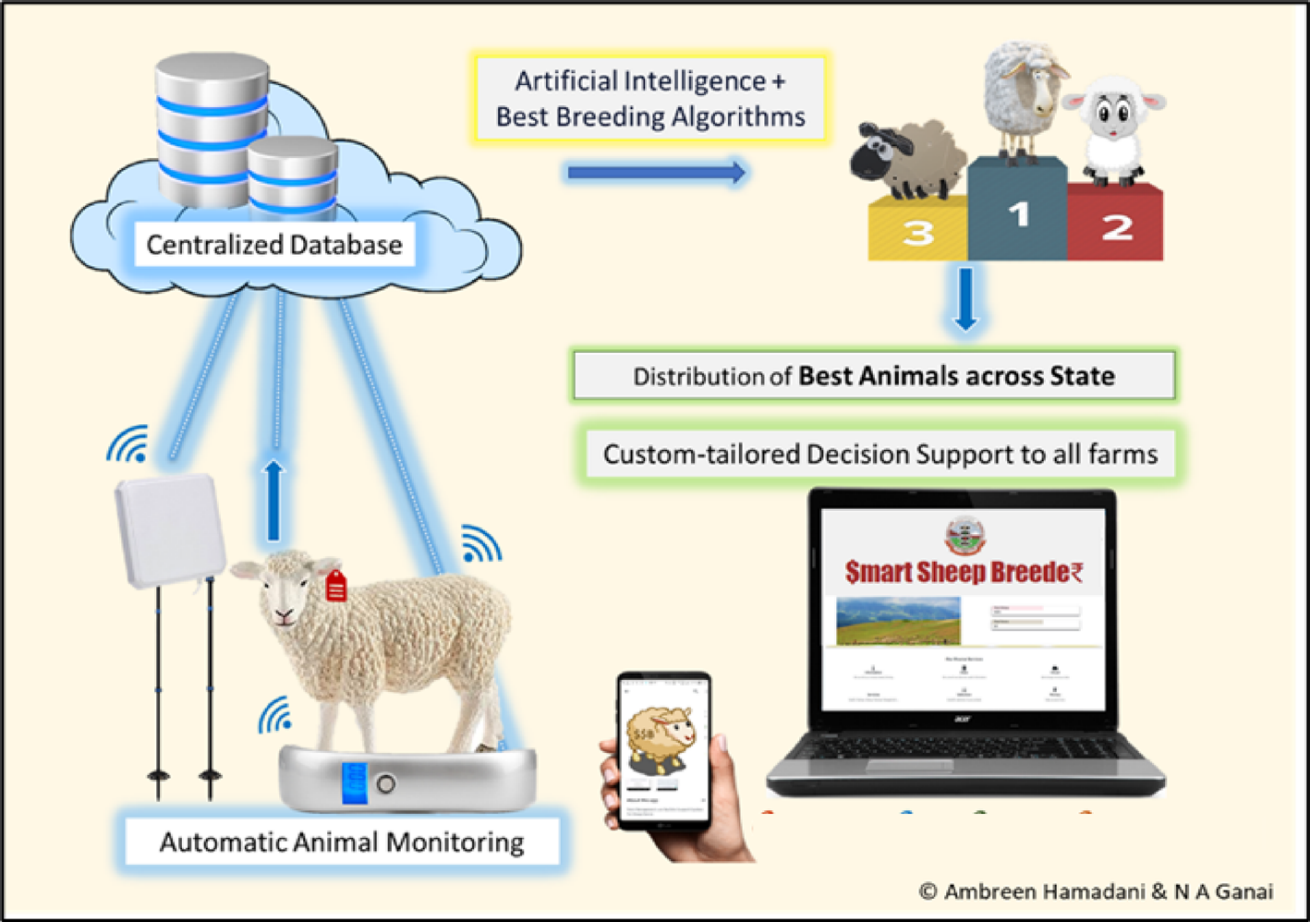
Smart Sheep Breeder and its potential.

**Figure 3:**
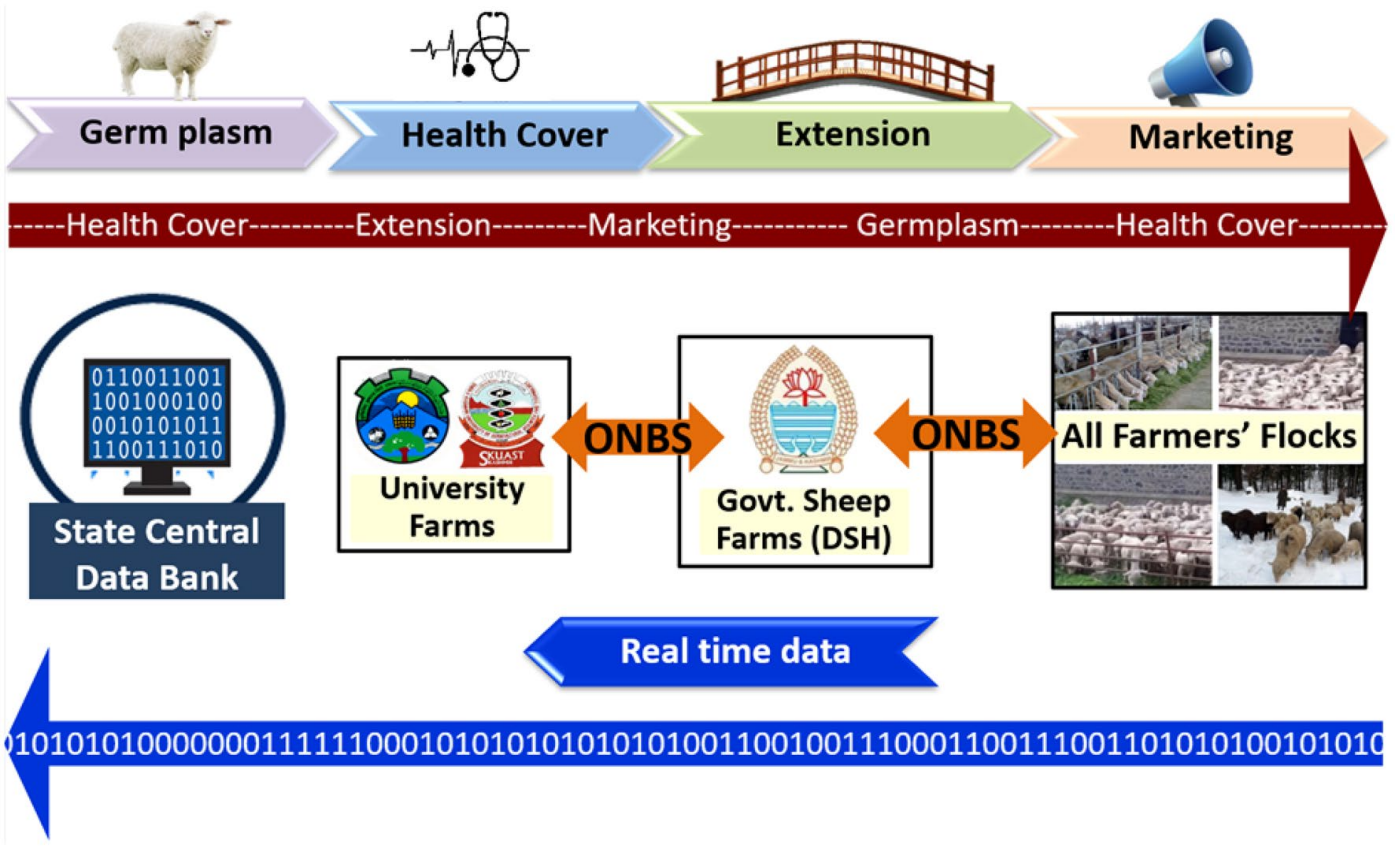
Smart Sheep Breeder for ONBS and data flow.

Smart Sheep Breeder—an artificial intelligence-driven fully functional, multi-use DSS for performance recording, farm data management, breeding, ranking of animals on genetic merit, and decision-making in sheep farms was developed (http://www.agbskuastk.org/smartsheepbreeder). An android application, “Smart Sheep Breeder” was also developed for the facilitation of data input and was hosted on Google Play. The potential of SSB is represented in Figs. 2 and 3.

The system is copyright protected in India (No: SW-13653/2020) and a patent for the same is also published (No: 202011035476). The tool has also been adopted into Jammu and Kashmir State livestock breeding policy operational plan, 2019^31^.

Personalized homepages for the website were also developed for redirection to various sections of the DSS. A log-in and registration system was also created.

**Figure 4:**
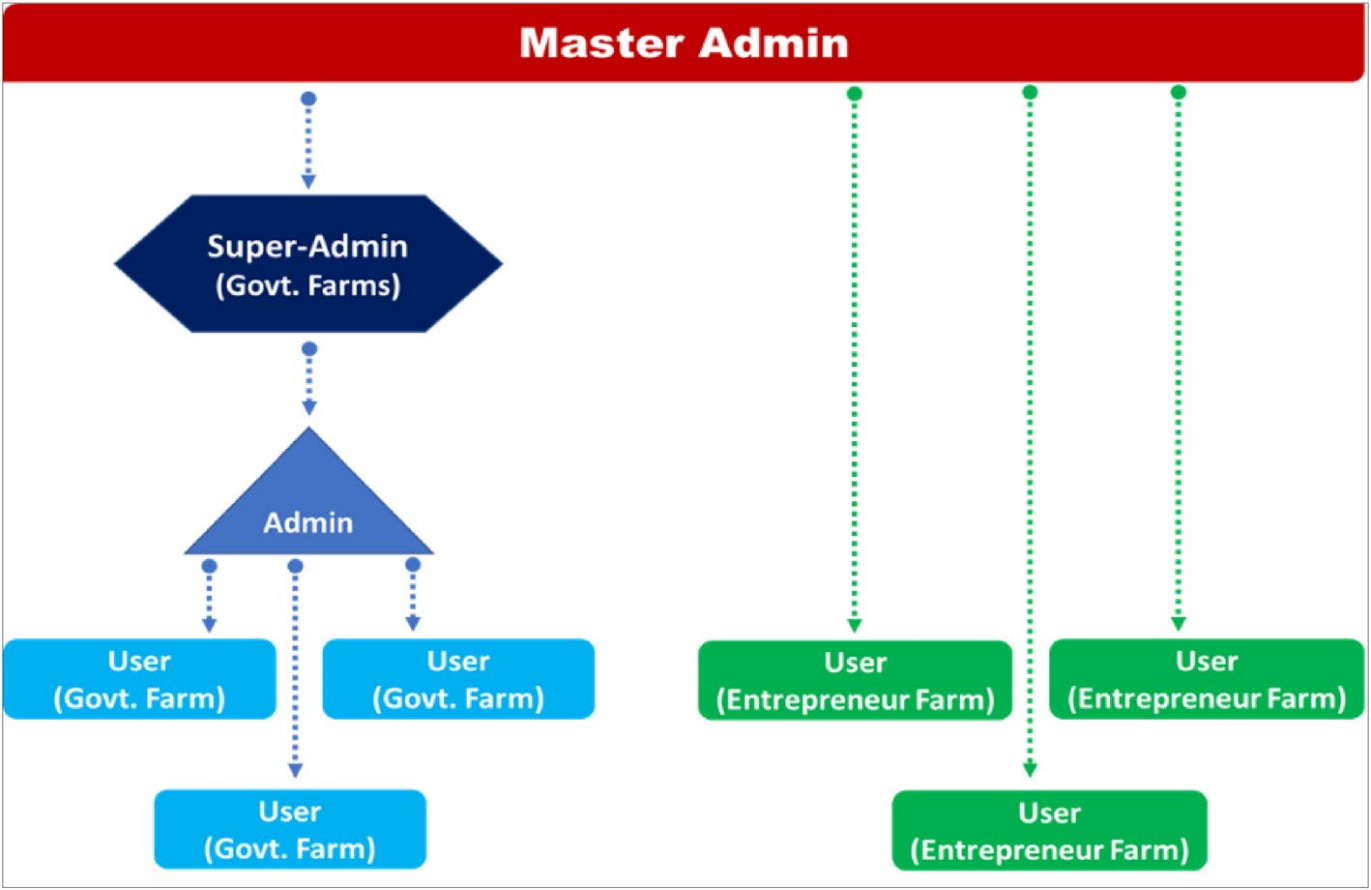
Framework used for the creation of user roles.

The present DSS was created to support the management of the content of different portals of the DSS so that customized content could be displayed according to user type which is given in Fig. 4. Administrators can view and analyze reports generated from all registered farms/users under their specific jurisdiction. The user types provided by our system are based on a hierarchy depending on the role of the person at the farm. The types of users include a super admin who can control all farms under his jurisdiction and has access to all data. Officials under him have access to the farm data of a lesser number of farms. Individual farmers can only access details about their farms.

The user may also view all reports generated for only his farm to ensure privacy. More than 40 types of reports and graphs are generated by the system automatically on any date (current or previous) for all breeds registered within the system. Screenshots of some reports generated are given in Annexure 1. These include reports regarding the following.

- **Farm Inventories** This portal includes reports about total sheep (breed-wise), total ewes (breed-wise), total rams (breed-wise).
- **Sheep Inventories** This portal includes various reports about ewes’ inventory, rams’ inventory, breedable ewes’ inventory, breedable rams’ inventory, and wether inventory. These inventories include lists of all animals that are/were present on the farm on any date including all relevant details about them like age, parturition history, etc.
- **Lambings and abortions** This portal has been created to include extensive reports about lambings and abortions, abortion inventory, lambing inventory, and all relevant details thereof.
- **Production reports** This portal includes reports about body weights (breed-wise), body weight inventory, and body measurements at various ages on any date.
- **Wool quality** This portal includes reports about wool production inventory including the number of clips and other details about the quality and quantity of wool from each animal.
- **Health** This portal includes reports about the disease, vaccination and deworming, disease prognosis, and the drugs used as well.
- **Selection and breeding** This portal includes reports about (individual breeding values, BLUP breeding values, inbreeding coefficients, selection index values, and an artificial intelligence-based module. The selection indices and breeding values can be calculated for multiple traits as per the requirements of the farm. These include both meat production traits and wool quality and quantity.
- **History** This portal includes reports about pedigree reports and comprehensive history sheets of individual animals including their production, performance, pedigree as well as progeny.
- **Disposal** This portal includes reports about total disposals (breed-wise), disposed of animals’ inventory, and animal death inventory. These include all details about the death, disposal, or transfer of animals to other farms.
- **Graphical analysis** This portal includes graphical reports displaying farm status or animal status and their trend over the years.

### Centralized database

The entire system was successfully integrated with a centralized relational mega database (MySQL) to consolidate all data. This would be useful in big data mining and ranking all animals across farms based on pure genetic merit (Fig. 2) as the number of farms associated with this tool increases.

### AI-based module

**Figure 5:**
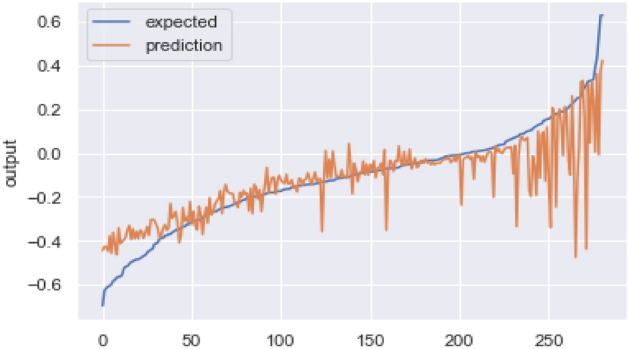
Correlation between true and predicted values for the AI algorithm.

The model developed for the prediction of breeding value quickly and in real-time had a testing correlation of 82.48. Figure 5 presents the correlation between true and predicted values from the test dataset of the ANN algorithm. This algorithm was deployed using the Django framework using Python. The testing mean-absolute-error, mean-squared-error, and root-mean-squared error values were 0.0762, 0.017, and 0.130, respectively. The module was deployed to automatically evaluate the genetic merit of all sheep on the farm at any date.

The total number of data points (rows) in the dataset after data cleaning and outlier removal was 2500. The total features used to train the final model were 5. Among others, the weight at 12 months and dam weight had the highest feature scores. The final model consisted of 4 layers with 10 neurons per layer with Tanh as the activation function. A learning rate of 0.001 with the Adam optimizer gave the best results. The model was run for 500 epochs with a batch size of 10 and a validation split of 20%.

### IoT system integration

The system was successfully integrated with a customized IoT, and RFID-enabled weighing system (IDTECH Solutions) for automatic animal identification, weight recording, animal monitoring, and counting of all RFID-tagged animals. The machine was installed, tested, and validated at SKUAST-Kashmir. All animals bearing RFID tags on their ears can be automatically captured by this system. The in and out antennae can record the animal entry and exit on the farm thus counting the animals and identifying them. The weighing bridge captures the timestamp, animal id, and weight which is recorded over the internet into the centralized database in real time. Additionally, Smart Sheep Breeder can be connected over the internet to any number of IoT-based data-gathering systems through the developed APIs. The APIs so created are for data recording of biometrical data about the flock.

### Integration, testing, and adoption

The results of heritability auto-generated by our system were validated using a standard example as per Cameron^21^. The book results were 0.38, and the results obtained by our system: were 0.37. The validation results of BLUP breeding values are given in Table 1. Validation of heritability using Wombat^23^ is given in Table 2.

**Table 1:** Breeding values validation.

Animal number	Breeding value smart sheep breeder	Breeding value^22^
1	0.01068661	0.011
2	− 0.00216870	− 0.002
3	− 0.05279482	− 0.053
4	− 0.09505783	− 0.095
5	0.09505783	0.095
6	− 0.06030577	− 0.060
7	0.06355883	0.064
8	− 0.05078197	− 0.050
9	0.12672115	0.127
10	− 0.15829755	− 0.158
11	0.02079067	0.021
12	0.05304874	0.053

**Table 2:** Comparison of heritability values with Wombat software.

	Birthweight	Weaning	6 Month	9 Month	12 Month
WOMBAT	0.346	0.206	0.163	0.130	0.159
R Code	0.354	0.220	0.166	0.136	0.156

**Figure 6:**
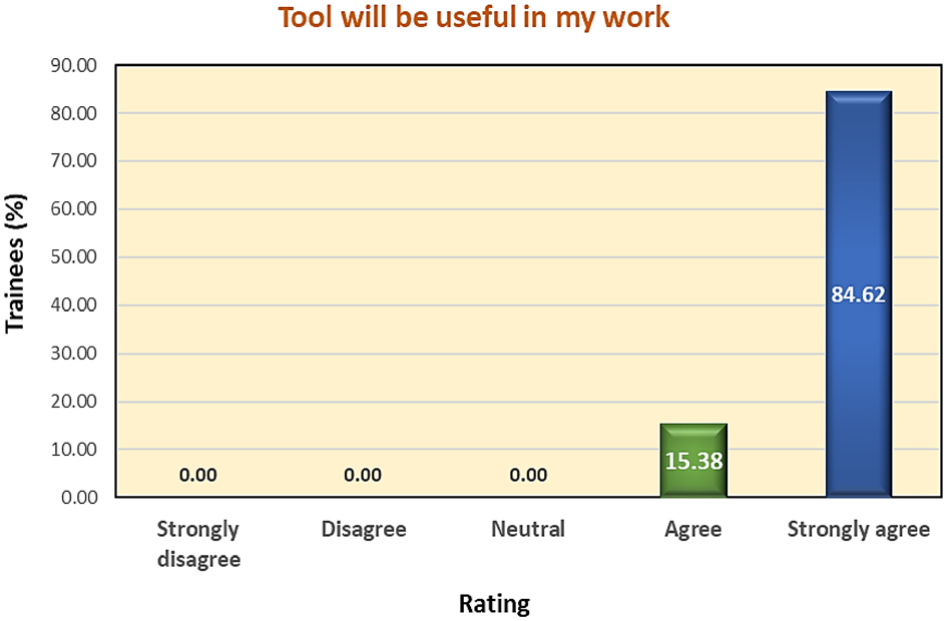
Response by users regarding the utility of the tool.

All 16 Government sheep breeding farms of J&K were registered for sensitization as well as beta testing of the tool. The feedback of the farm managers/officers of sheep husbandry departments indicated that 100% of the respondents were satisfied and agreed upon the usefulness of the tool in their work (Fig. 6) and that the developed tool was the need of the hour (Table 3). Since the tool is capable of farm data collection, analysis, interpretation, breeding value estimation, and ranking of animals based on genetic merit automatically and was found useful by the stakeholders, the hypothesis was successfully tested.

**Table 3:** Beta testing results.

S. no.	Agreement level	DSS considered useful in their work (%)	DSS considered the need of the hour (%)	Satisfied with the tool (%)
1	Strongly agree	84.62	84.62	69.23
2	Agree	15.38	15.38	30.77
3	Neutral	00.00	00.00	00.00
4	Disagree	00.00	00.00	00.00
5	Strongly disagree	00.00	00.00	00.00

## Discussion

Efficient farm management, good scientific research, and effective selection are possible only if the data are easily accessible, accurate, and gathered routinely. The upkeep of paper records is not only cumbersome and difficult to maintain but is also hard to replicate and requires a lot of physical space. The lack of proper digitized farm records has hindered farm development as well as the creation of future-oriented breeding policies. Also, the absence of a centralized cloud-based database and the unavailability of duplicate records leads to the possibility of data loss due to factors like pests, theft, loss, fire, floods, etc. which have happened in some instances in the past. Progress in the farm operation cannot be determined from year to year without keeping an inventory and deriving rapid inferences from paper records is impractical but a digital inventory is flexible, efficient, rapid, and accurate^32^. A web-based DSS catering to most aspects of sheep farm data management would help in the creation of a system for the safe storage, analysis, retrieval, and effective use of data.

It is almost impossible to draw real-time, on-spot inferences from manual data. This especially hinders breeding decisions that require procedures that are computationally expensive and require vast amounts of data. Having to manually enter massive amounts of data into the system before analysis consumes a considerable amount of a researcher’s time and energy^33^. As a result, research is not only delayed but the quality of the actual research is compromised. Therefore, selection on farms in India is still intuition based and the genetic progress is very low. The benefits in terms of saving time by efficient data handling have also been stressed by Lawson et al*.*^34^.

E-linking of the University farm with other sheep farms would be necessary for ONBS which would generally increase the genetic merit of animals across farms^35^. Web-based farm management information systems facilitate internet-based collaborative research by connecting geographically dispersed farms with experts or customizing end-user data for analysis or presentation^36^.

As also suggested by Fountas et al*.*^37^ agriculture has entered a new era in which the purpose of a DSS is to meet the increased demands, reduce production costs, and increase the overall productivity of the farm^38^. Government farm managers and officers of the Sheep Husbandry Department had felt the need for this tool and their complete agreement upon sensitization in its utility enforced the hypothesis of this research. The key to success is access to timely information and elaborate decision-making. Decision-making is an important aspect of farm management as well as e-governance. Similar tools for dairy cattle have been developed^8^. The most popular one among them is INAPH, launched countrywide by the Government of India. Commercially available superior quality tools for sheep farm management were found to be scanty and inadequate. Additionally, they are complex and difficult to use^39^ and are not applicable to farm practices and present systems of data collection on Indian farms. Adoption of such systems would force such farms to alter some of their processes to fully utilize the product thus adding to the problem rather than subtracting from it^40^. This would also reduce adoption rates substantially. SSB solves this issue by being custom tailored and specifically designed for challenges faced by farmers in India.

Additionally, the available data management software capable of estimating genetic parameters for ranking animals according to their genetic merit is not easily available and those that are, do not assist in making breeding-related decisions. This necessitated the need for the development of a custom-tailored and multi-dimensional DSS for catering to the needs of the sheep farms. Economic, as well as scientific progress, could be ensured by adopting the present tool because it is simple to use, easy to understand, and cheap. Also, the present tool was developed with a user-friendly interface for sheep farms. Automated data processing would also facilitate decision-making in real-time, futuristic, and effective policy planning for breeding by administrators, and scientific research on the farm and allow the integration of expert knowledge and farming preferences for the benefit of both. Managemental strategies could be improved using this tool through proper interpretations of the trend/status of the farms. Smart Sheep Breeder is a step toward precision animal farming which has also been reported to increase efficiency, reduced labor and improve overall animal well-being^41^.

With the University as a stakeholder, farmers would generally be more comfortable with sharing data than with large companies that may repurpose the data for corporate interests. This is also a primary concern of India’s digital agriculture mission.

The success of animal farming in developed countries may mainly be attributed to the incorporation of specific DSS and breeding tools. Sheep Genetics Australia, the National Genetic Information and evaluation services for the meat and wool sector, delivered as LAMBPLAN, MERINOSELECT hosts a database of about 6.5 million animals and more than 1000 flocks in Australia and overseas.

Roles assigned to specific users ensure that relevant data is served to the users as per their role and ensure privacy for farmers. For e-governance purposes i.e., for the remote monitoring of all farms under his jurisdiction, an administrator needs to have access to data across all farms to get a general overview of all farms. He/she could thus remotely monitor all farms under the jurisdiction, visualize the farming trends in real-time and be able to take effective data-backed decisions. All this must be achieved without compromising the accuracy of the records from other farms thereby maintaining the privacy and hassle-free use of the web-based tool.

The most important function of a DSS is to handle and benefit from enormous data volumes and convert raw data into useful information^39^. These would give the farm manager an understanding of the farm situation and the administrator to take timely critical decisions related to all aspects of farm management. Farm reports serve as an aid to managerial control during production. A producer can keep track of events such as whether activities are going according to plan, the total strength of the farm in terms of animals according to their sex and age, when animals were vaccinated, dipped, given any medicine, or castrated, which breed on the farm is performing better and which breed is not and production from animals both in terms of quality and quantity may be effectively monitored. Inferences drawn from disposal reports may give an early indication of any potential problems at the farm e.g., epidemic, managemental errors, etc. An analysis of reports related to breeding may help the breeder to find out expected dates of parturition, help in removing sterile/infertile animals, and make use of superior quality rams for breeding. History sheets of individual animals provide all information about that animal which helps the breeder to draw inferences and make decisions regarding breeding/culling/sale of the animal.

A breeder may also trace the pedigree of animals and serve as a tool for the selection of breeding animals and refrain from mating animals that are closely related to each other. A sound breeding program can only be developed by understanding which pedigree to use based on the information collected about each ancestor.

The tool was validated by both alpha and beta testing. The similarity of the results obtained by the DSS with book values and standard tools proves its accuracy. The retrospective data was used so that the tool could be validated quickly. Since the tool was developed to eventually be used on real-world farms, actual farm data, both real-time as well as retrospective was used to validate the tool. The results produced by the system could thus be compared with true data and they were found to be accurate. E.g., The lambing rate, animal parities, disposal rates, and farming trends were found to be 100% accurate. Results that could not be checked against farm records e.g., breeding values estimations were compared with standard methodologies or standard software like Wombat. The alpha testing and the feedback obtained through beta testing results validated the initial hypothesis.

The use of AI and various biometrical techniques for the ranking of animals through estimated breeding values and selection indices shall enable farm managers to retain only animals of high genetic merit on the farm and cull animals of low genetic merit and thus ensuring that genetic merit is maintained at the farm (Fig. 3).

The estimation of breeding values using the mixed model method for BLUP provides a powerful and flexible tool. Artificial Intelligence is also becoming a significant change in the agricultural sector. Deep learning models have also been used for sheep behavior recognition^42^. Lopes et al.^43^ also showed that the ANNs performed better than other ML techniques for the prediction of genetic merit. AI is also finding applications in wide-ranging areas of research^44,45^ including systems for providing decision support^46^.

A higher correlation of 0.89 than that obtained by the present study, using ANN was reported for breeding values prediction by Bangar et al.^47^ in the Harnali sheep breed. Ghotbaldini et al.^48^ for the prediction of breeding values in Kermani sheep also used two ANN models and reported correlation coefficients of 0.703 and 0.864. Our results are also consistent with the reports of other scientists in this area^49,50^. Also, a low learning rate was found to give better results than a higher one by Brownlee^51^ because high learning rates may cause unstable training and the neural network may never actually converge. IoT-based tools are rapidly becoming a norm in the world^52^. This is true also for the animal sciences. Cheng et al.^42^ also suggest the use of sensors, and machine learning, monitoring for monitoring animals and farms through automation which is necessary as farming is becoming more and more intensive. The AI model used would enable the farmers to know the genetic superiority of their animals at their fingertips and therefore make the right breeding decisions.

There are no major ethical issues involved with the adoption of this tool as no animal interventions are performed by this. Additionally, the IoT compatibility of the system is to ensure that animal handling is minimized and that the drudgery of data entry is eliminated. Most farms in J&K today are based on manual weighing systems which are labor-intensive and involve a tedious ledger-based record-keeping system. The development of a technology-driven system for farm management would promote both farm and animal welfare (as the animals do not need to be lifted by a rope to be hung from the spring balance for weighing anymore). The ultimate goal of developing this system is to minimize handling animals which would reduce animal distress and as farming becomes resourceful, more animals could be raised by a single farmer without additional labor.

A major threat to validity is the use of spurious data. This has been prevented for the weight records by the use of IoT-based data entry operations. In addition, the tool uses various checks like limiting entries to format types or setting upper and lower limits for value entries to ensure that outlier data is not entered into the system. This could be further improved by using AI for data validation as well. The threat of low adoption has been mitigated to a large extent by ensuring the system is simple to use and easily accessible. Also, the steady penetration of the internet and mobile phones to remote corners of the Country would help in the popularization of the tool. This is also in line with the Digital India mission of the Govt. of India.

BLUP has become the worldwide standard for the prediction of breeding values of farm animals. This is because, this method minimizes the variances of errors, the correlation between predictors and predictions are higher, the probability of selecting the better of any pair of candidates is maximized and the expected mean of the breeding values of selected individuals is maximized. The DSS also generates inbreeding coefficients like a tool reported by^53^, which may help the farm manager to monitor inbreeding on the farm and make necessary decisions regarding the import of new animals into the farm or culling of inbred animals whenever required.

The generated report on selection index values of all live animals on the farm for the DSS shall serve as a breeding tool to make the selection on more than one trait at once. Since in practice, the index value is a weighted BLUP-EBV of selection candidates, therefore, the complexity of b = P–1Gv was avoided. The weighted sum of EBV and (economic) breeding goal weights for the construction of an index are also used in the SelAction tool, LAMBPLAN, and WOOLPLAN as reported by^27^. A single selection index may not serve the interest of all farms because each producer may have different feed costs and sales markets so an index specifically for that flock would be better than a one-size-fits-all index.

## Conclusion

Smart Sheep Breeder is an AI-based, and IoT-compatible decision support system catering to most aspects of sheep farm data management. It can consolidate data across all farms into a centralized database for ranking animals on pure genetic merit and dissemination of elite germplasm. This would help in the genetic improvement of sheep, animal welfare, and farmer welfare as well as improving the farmer’s income by increasing the efficiency of the farm.

## Data Availability

The datasets used for the validation of the tool are not publicly available due to restrictions on the public availability of the data but are available from the corresponding author on reasonable request.
